# Prognostic significance of preoperative serum tumor markers in hepatoid adenocarcinoma of stomach (HAS)

**DOI:** 10.1186/s12885-023-10516-y

**Published:** 2023-01-16

**Authors:** Xuesong Yang, Anqiang Wang, Jialin Li, Kai Zhou, Ke Ji, Xin Ji, Ji Zhang, Xiaojiang Wu, Zhaode Bu

**Affiliations:** Key Laboratory of Carcinogenesis and Translational Research (Ministry of Education/Beijing), Center of Gastrointestinal Cancer, Peking University Cancer Hospital & Institute, Beijing, China

**Keywords:** Gastric hepatoid adenocarcinoma, Alpha-fetoprotein, Carbohydrateantigen19–9, Prognosis

## Abstract

**Background:**

The role of preoperative serum tumor markers in HAS patients was vague, we designed the study to explore the effect of preoperative serum tumor markers on predicting the prognosis of HAS patients.

**Methods:**

A total of 139 patients were included according to the different tumor makers. X-tile tool was employed to identify the optimal cut-off values of respective tumor makers. Multivariate analyses were conducted to determine independent risk factors.

**Results:**

The optimal cut-off value of alpha-fetoprotein (AFP) for 3-years overall survival (OS) and recurrence-free survival (RFS) was 516 ng/mL. Patients with high-level AFP values assumed significantly worse OS and RFS than those with low-level AFP values (*P* = 0.028 and *P* = 0.011, respectively). The optimal cut-off value of Carbohydrate antigen (CA)19–9 for OS and RFS was 51.3 U/mL. And the survival results were similar with AFP in the aspects of OS and RFS (*P* = 0.009 and *P* < 0.001, respectively). Multivariate analyses showed that high serum AFP was an independent risk factor for OS and RFS of HAS patients (HR7.264; 95% CI 1.328–39.738; *P* = 0.022 and HR 2.688; 95% CI 0.922–7.836; *P* = 0.070, respectively). CA19–9 could perform as a fair substitute to predict the HAS patients’ OS and RFS when the preoperative serum AFP was unavailable (HR 7.816; 95% CI 2.084–29.308; *P* = 0.002 and HR 4.386; 95% CI 1.824–10.547; *P* = 0.001, respectively). Other tumor markers didn’t present significant influences.

**Conclusions:**

Applying preoperative serum AFP level to predict the HAS patients’ prognosis is feasible and preoperative serum high-AFP is an independent risk factor for OS and RFS of HAS patients. Preoperative serum CA19–9 could be an alternative choice when AFP was absent.

## Introduction

Gastric cancer is one of the most common malignancies which ranks fourth in morbidity and second in mortality in China [1] . Hepatoid adenocarcinoma, as a special pathological type of gastric cancer, is characteristic by the serum high AFP which is frequently elevated in patients with hepatocellular carcinoma (HCC) [2]. The elevated serum AFP values, like HCC, is a prominent trait of HAS. Compared to the common gastric adenocarcinoma, HAS presents a poor prognosis for the high rates of lymph nodal and distant metastasis [3–5]. HAS accounts for 0.3–1.0% of all types of gastric cancer, which results in the under-recognized of HAS [6, 7]. Being a common method for screening and surveilling the recurrence of the tumor, the examination of serum tumor markers is accessible on a large scale. Serum tumor markers such as AFP, Carcinoembryonic antigen (CEA), CA19–9, CA72–4 and CA24–2 already used to assess the biological behavior and progression of gastric cancer [8]. The analyses of relative tumor markers may prompt our awareness of HAS, affect treatment decisions and improve the prognosis of patients with HAS.

The relations of high AFP and gastric cancer were widely acknowledged owing to the previous works. Chen et al. analyzed 1286 patients with gastric cancer and suggested that high level of serum AFP was significantly linked to the poor survival of gastric cancer [9]. However, the relation of high serum AFP and survival of HAS patients are still vague. To date, no large-scale studies have been conducted to research the prognosis significance of high serum AFP among HAS patients, not to mention the quantitative analyses. In addition, the roles of other tumor markers like CA19–9, CEA, CA72–4 and CA24–2 played in HAS are controversial.

Therefore, we conceived our study to explore the significance of serum tumor markers in the prognosis of HAS.

## Materials and methods

### Patient selection

we included patients with HAS who underwent the gastrectomy form October 2009 to July 2021 at the Center of Gastrointestinal Cancer of Peking University Cancer Hospital. All patients enrolled in the study were Chinese. Patients in the cases of the following would be excluded: existing intraperitoneal metastasis cancer (including Peritoneal metastasis and positive cytology rather than suspicious positive cytology) before the operation, not achieving R0 gastrectomy and unavailable pre-treat tumor makers data. All clinical data and materials collection were given informed consent from patients.

### Disease management

All HAS patients in the study were clinically confirmed by gastroscopic biopsy and histological examination. Computed tomography (CT) was also necessary for helping surgeon determine the tumor location and border, identify the lymph node stage and distant metastasis. Serving as an auxiliary diagnostic tool, tumor markers examination was often performed within 2 weeks before the gastrectomy. By the way, AFP was added to the routine tumor markers examination panel to avoid misdiagnosis and missed diagnosis of HAS Since 2016 and CA24–2 was not the routine tumor markers examination so far. Therefore, some AFP and CA24–2 data were absent.

Whether undergoing preoperative treatment (neoadjuvant chemotherapy) was based on the recommendations of The National Comprehensive Cancer Network (NCCN) guideline [10] and patients’ willing. Resectability and surgical resection protocol was formulated by a multidisciplinary team conference after communication with patients. The strategy of postoperative treatment was determined by the pathological evaluation [11]. S-1 and oxaliplatin based chemotherapy regiment was frequently used [11].

### Definitions

**Fig. 1:**
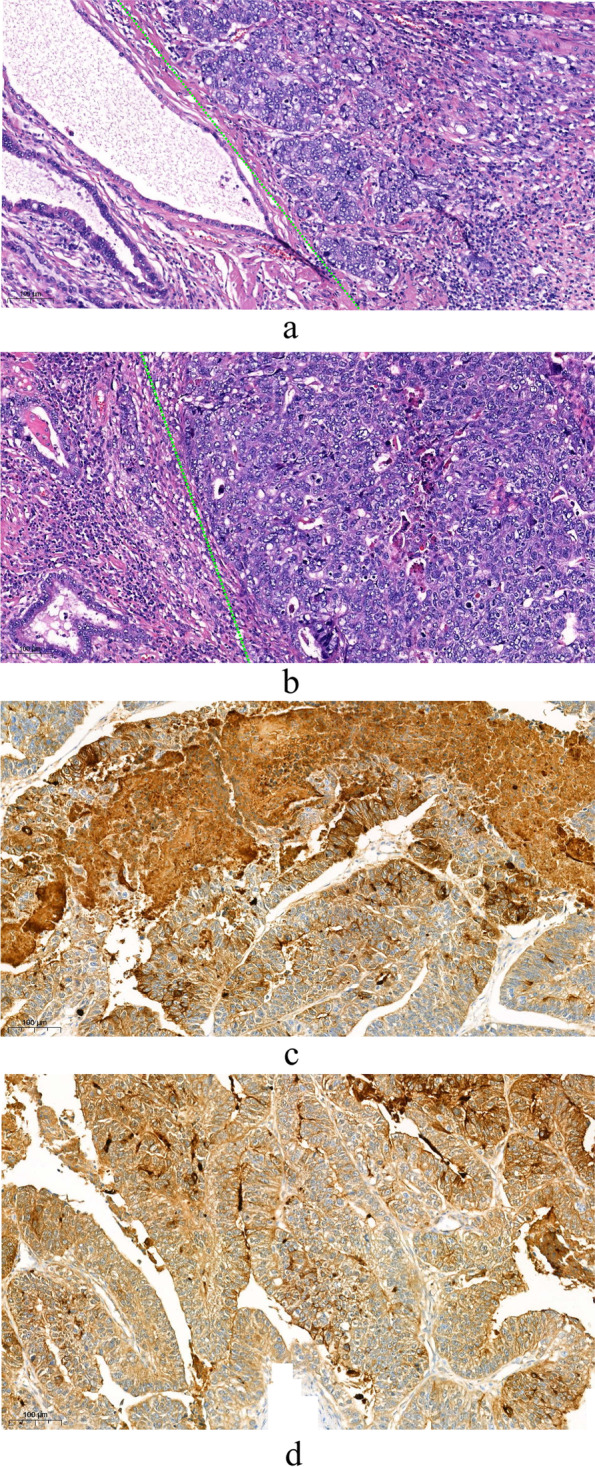
Pathological pictures of typical hepatoid adenocarcinoma of the stomach (HAS). In Figure **a** and **b**, the hepatocellular differentiation area was showed on the right part and adenocarcinoma area was showed on the left part. If the hepatocellular differentiation area was found in the primary gastric cancer tissue after gastrectomy, the patients would be diagnosed as HAS in this study. Figure **c** and **d** showed two typical positive AFP stain section.

The diagnostic criteria of HAS in the study was the pathological “gold standard” that the area of hepatocellular differentiation was found in the primary gastric cancer tissue after gastrectomy, no matter the level of AFP value [6, 12, 13]. Patients would be diagnosed as HAS once the area of hepatocellular differentiation was detected. The two typical pathological pictures were exhibited on Fig. 1 a and Fig. 1 b. The hepatocellular differentiation area (right part) and adenocarcinoma area (left part) was showed in the same pathological section. AFP immunohistochemical stains was used in the histopathological diagnosis in this study (Fig. 1 c and Fig. 1 d). R0 resection was defined as pathologically negative margin≥1 mm [14]. The indicators of OS and RFS were regarded as the major end point. OS refers to the time from first treat (neoadjuvant chemotherapy or surgery) to death caused by any reason or the last follow-up, and RFS refers to the period from gastrectomy to the tumor recurrence or the last follow-up.

### Surveillance and follow-up

The examination of surveillance including abdominal and pelvic CT and tumor markers test would be performed to identify whether the tumor recur during follow-up. The patients would under the first follow-up in the 1 month after the gastrectomy. Then every 3 months follow-up would be performed in the next year. In the second and the third year after the gastrectomy, the follow-up would be conducted every 6 months. Telephone follow-up call was the major form.

### Statistical analyses

The indicators of OS and RFS were regarded as the primary endpoint and the second endpoint respectively. If normally distributed, continuous variables would express as mean ± standard deviation(SD) and be compared by the Student’s t test, otherwise continuous variables would express as medians with interquartile range(IQR) and be compared by the Mann-Whitney *U* test. As for categorical variables, if they were ranked data, they would be compared using Mann-Whitney *U* test, otherwise they would be compared by Pearson’s chi-square test or Fisher’s exact test determined by the sample size. The optimal cut-off values of AFP and CA19–9 were determined by X-tile analytical tool [15]. Survival analyses including RFS and OS were estimated by Kaplan-Meier method (Kaplan & Meier, 1958) and the inter-groups difference were judged by log-rank test. If the risk factors were significant statistically in univariate analyses (*P* < 0.050), they would be recruited in multivariate Cox regression analyses to confirm the independent risk factors influencing RFS and OS with the widely accepted significant factors. The X-tile analyses used X-tile tool software® (Yale university 2003–05, USA; version 3.6.1). Statistical analyses used SPSS® (IBM Corp., USA, version 26) and relative curves were plotted by GraphPad (GraphPad software, USA). If *P* < 0.050 (or *P* < 0.100 in multivariate analyses), the differences would be deemed as significant statistically.

### Ethics approval and consent to participate

This study has received the approval of the Ethics Committee of Peking University Cancer Hospital and Institute. Informed consent was obtained for all participants.

## Results

### The patients enrollment

**Fig. 2:**
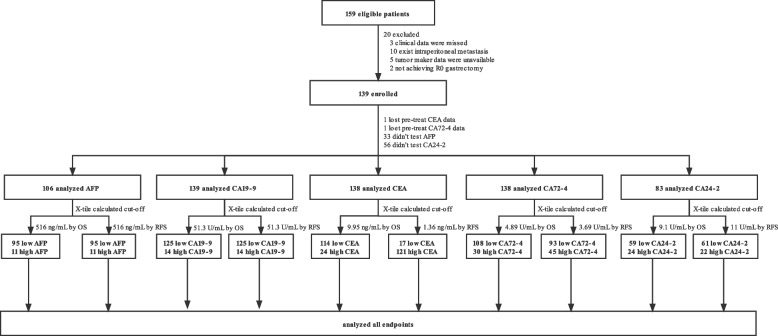
Flow diagram. A total of 159 patients were included based on inclusion criteria and then 20 patients were excluded based on exclusion criteria. Eventually, 139 patients were enrolled in the study. Subsequently, we analyzed specific tumor markers by X-tile calculating and survival analyses using respective patients’ data. AFP, alpha-fetoprotein; CA19–9, carbohydrate antigen 19–9; CEA, carcinoembryonic antigen; CA72–4, carbohydrate antigen 72–4; CA24–2, carbohydrate antigen 24–2; OS, overall survival; RFS, recurrence-free survival

Form October 2009 to July 2021, 159 patients pathologically diagnosed with HAS underwent gastrectomy were enrolled in our study at the Center of Gastrointestinal Cancer of Peking University Cancer Hospital. A total of 20 patients were excluded on the basis of exclusion criteria. Three patients had no enough clinical data, 10 patients with intraperitoneal metastasis cancer before the operation, 5 patients had no pre-treat tumor maker data and 2 patients did not achieve R0 gastrectomy. Ultimately, 139 patients were included in our study. Particularly, 2 out of them lost pre-treat CEA data and CA72–4 data, respectively. 33 out of them failed to test AFP and 56 out of them didn’t test CA24–2 before the surgery. Therefore, in order to take full advantage of the patients’ clinical data, we decided to analyses specific tumor markers using respective patients’ data. (Fig. 2).

### Determinant of the optimal serum tumor markers cut-off values

We employed X-tile tool to find out the optimal cut-off value of tumor markers.

**Fig. 3:**
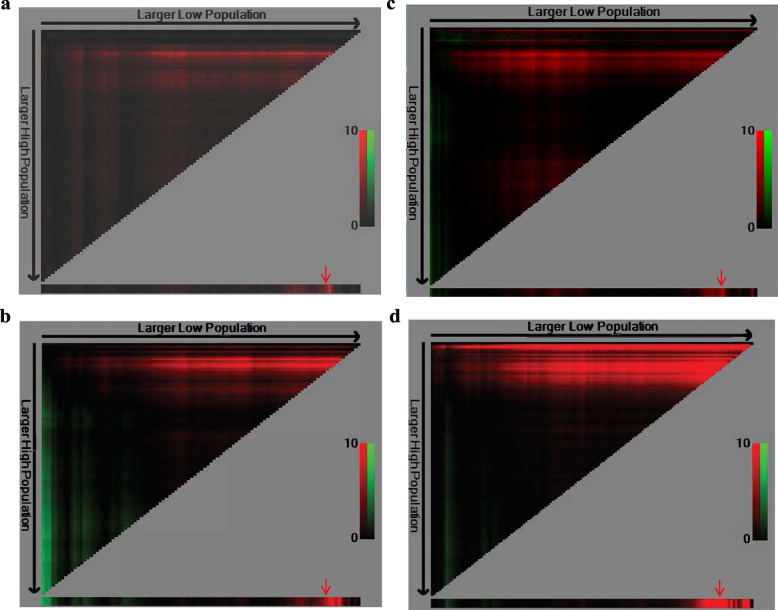
X-tile profile. X-tile was employed to calculate the optimal cut-off values using respective patients’ data. The optimal cut-off point marker by red arrow. 516 ng/mL of AFP was the optimal cut-off value for 3-year OS(**a**) and RFS (**b**). 51.3 U/mL of CA19–9 was the optimal cut-off value for 3-year OS (**c**) and RFS (**d**). AFP, alpha-fetoprotein; CA19–9, carbohydrate antigen 19–9; OS: overall survival; RFS, recurrence-free survival

The optimal cut-off values determined by X-tile tool were the points where differences across groups exhibited the most significant for 3-year OS or RFS. In 106 patients whose AFP data were available, 516 ng/mL was the optimal cut-off value for 3-year OS (maximum χ2 log-rank values 4.841) and RFS (maximum χ2 log-rank values 6.510). In 139 patients whose CA19–9 data were available, 51.3 U/mL was the optimal cut-off value for 3-year OS (maximum χ2 log-rank values 6.796) and RFS (maximum χ2 log-rank values 15.457). In 138 patients whose CEA data were available, 9.95 ng/mL was the optimal cut-off value for 3-year OS (maximum χ2 log-rank values 2.855) and 1.36 ng/mL was the optimal cut-off value for 3-year RFS (maximum χ2 log-rank values 4.961). In 138 patients whose CA72–4 data were available, 4.89 U/mL was the optimal cut-off value for 3-year OS (maximum χ2 log-rank values 0.806) and 3.69 U/mL was the optimal cut-off value for 3-year RFS (maximum χ2 log-rank values 3.622). In 83 patients whose CA24–2 data were available, 9.1 U/mL was the optimal cut-off value for 3-year OS (maximum χ2 log-rank values 1.218) and 11 U/mL was the optimal cut-off value for 3-year RFS (maximum χ2 log-rank values 3.095). In addition, we defined high-level as the value above or equal to the cut-off point and low-level as the value below the point. (Fig. 3).

### Clinicopathologic characteristics

Of 106 patients for AFP analyses, 95 cases fell into low AFP group and 11 fell into high AFP group. Beyond expectations, the differences across two groups were not significant in the aspects of gender, age, BMI, history of tumor, T stage, N stage, pathological stage, cycle of perioperative chemotherapy, location of tumor, Borrmann types, Lauren classification, surge type, vascular tumor thrombus and neural invasion (Table 1).

**Table 1:**
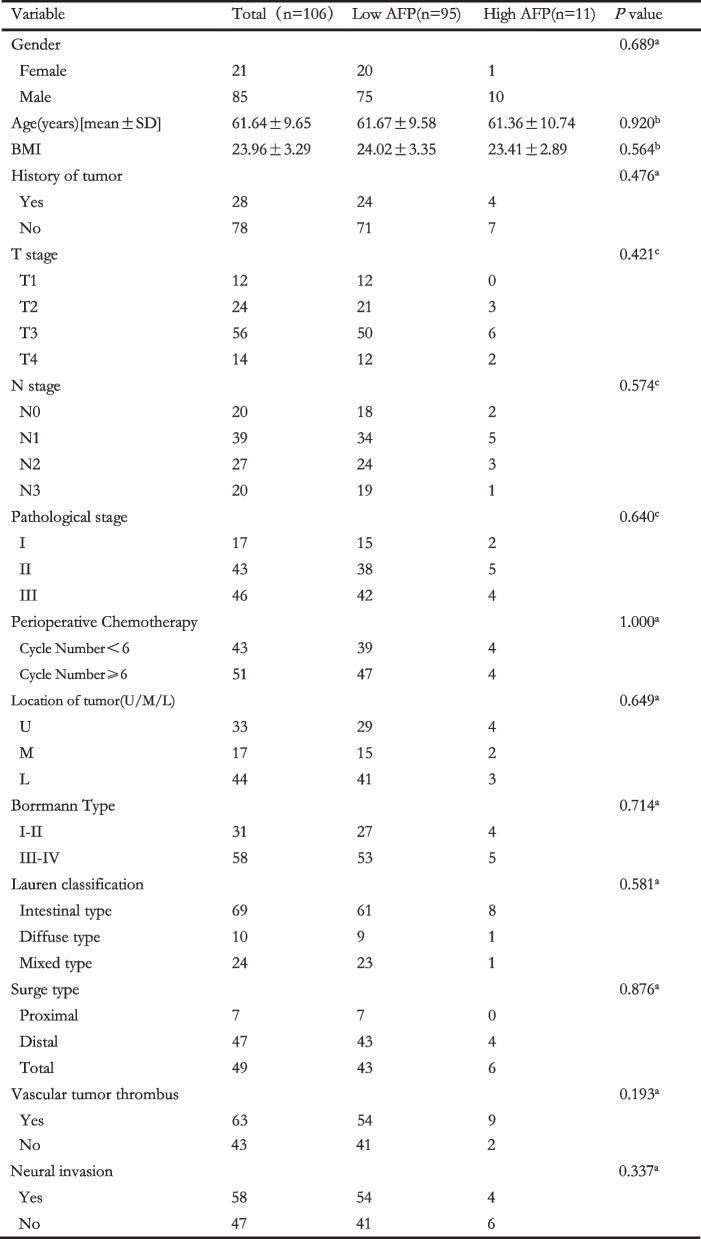
Clinical characteristics of patients with high- and low-level AFP

In the group of patients for CA19–9, 125 patients fell into low CA19–9 group and 14 fell into high CA19–9 group. The pathological stage and Borrmann types showed significant differences between two groups. In low CA19–9 group, 22 patients were stage I, 52 patients were stage II and 51 patients were stage III. In high CA19–9 group, 0 patient was stage I, 4 patients were stage II and 10 patients were stage III. We use the Mann-Whitney *U* method to test *P* value and the result was 0.018. In low CA19–9 group, 36 patients were Borrmann I/II types and 68 patients were Borrmann III/IV types. And in high CA19–9 group, 0 patient was Borrmann I/II types and 11 patients were Borrmann III/IV types. And the *P* value was 0.018 analyzed by Fisher’s exact test. However, there were no prominent differences between two groups in gender, age, BMI, history of tumor, T stage, N stage, cycle of perioperative chemotherapy, location of tumor, Lauren classification, surge type, vascular tumor thrombus and neural invasion (Table 2).

**Table 2:**
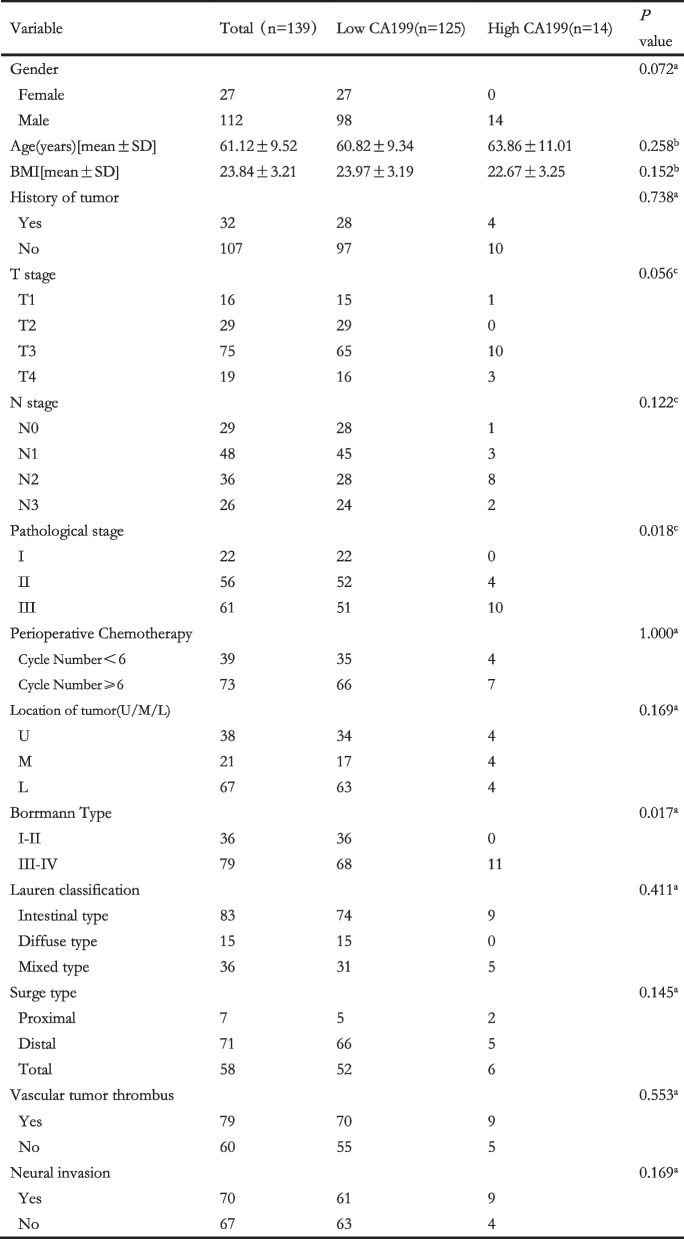
Clinical characteristics of patients with high- and low-level CA199

In addition, the clinicopathologic characteristics didn’t show any differences in analyzing CEA, CA72–4 and CA24–2 patients. we didn’t show their clinicopathologic characteristics because they fail to predict survival in our later analyses.

### AFP and CA19–9 could significantly predict survival of patients with HAS

**Fig. 4:**
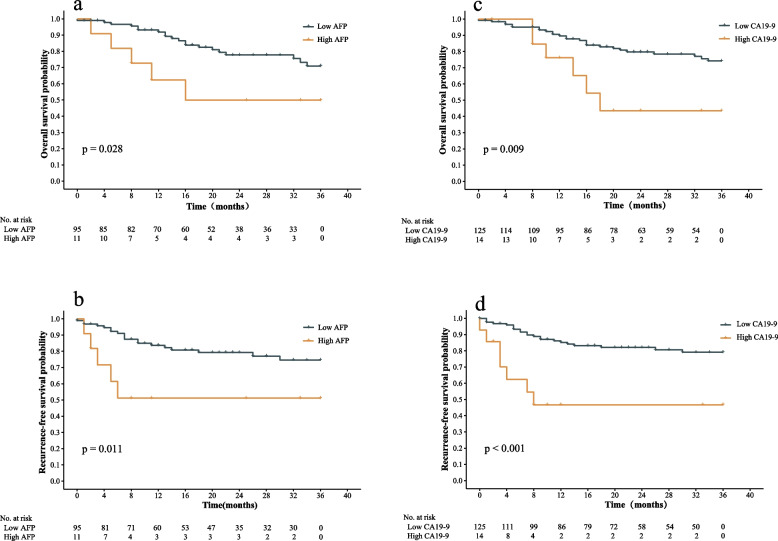
Survival analyses results. survival analyses of patients with low- and high-level AFP and CA19–9. The OS(**a**) and RFS(**b**) in the low AFP group were better than those in the high AFP group. Similarly, the OS(**c**) and RFS(**d**) in the low CA19–9 group were better than those in the high CA19–9 group. AFP, alpha-fetoprotein; CA19–9, carbohydrate antigen 19–9; OS: overall survival; RFS, recurrence-free survival

Survival analyses indicated that AFP and CA19–9 could functioned as an index to predict the prognosis of HAS patients. We applied Kaplan-Meier method performing survival analyses for the perspective tumor markers. In the AFP group, the differences were significant between two groups for OS (mean OS: 30.8 m vs. 22.5 m, *P* = 0.028) and RFS (mean RFS: 29.7 m vs. 20.1 m, *P* = 0.011). Similarly, in the CA19–9 group, there was also significant differences between two groups for OS (mean OS: 30.9 m vs. 23.0 m, *P =* 0.009) as well as RFS (mean RFS: 30.7 m vs. 18.9 m, P<0.001). (Fig. 4).

However, the OS differences were not significant in CEA (mean OS: 30.9 m vs. 25.9 m, *P* = 0.091), CA72–4 (mean OS: 29.6 m vs. 32.1 m, *P* = 0.369) and CA24–2 groups (mean OS: 31.3 m vs. 28.8 m, *P* = 0.270). Similarly, the RFS differences were also similar in CEA (mean RFS: 24.2 m vs.30.2 m, *P* = 0.026), CA72–4 (mean RFS: 30.9 m vs. 26.4 m, *P* = 0.057) and CA24–2 groups (mean RFS: 31.1 m vs. 25.1 m, *P* = 0.079).

To identify the independent risk factors, we performed Univariate and multivariate analyses. Univariate analyses indicated that BMI, cycle number of perioperative chemotherapy, Borrmann types, serum AFP level and CA19–9 level were closely associated with OS. Furthermore, multivariate analyses confirmed that AFP level was an independent risk factor of OS (hazard ratio [HR] 7.264; 95% confidence interval [CI] 1.328–39.738; *P* = 0.022). Other independent risk factors included BMI (HR 1.377; 95% CI 1.048–1.810; *P* = 0.022), cycle number of perioperative chemotherapy (HR 0.273; 95% CI 0.064–1.157; *P* = 0.078), Borrmann types (HR 12.283; 95% CI 1.131–133.414; *P* = 0.039). As for RFS, univariate analyses manifest that pT stage, pathological stage, and serum AFP level and CA19–9 level were prominently associated with RFS. And multivariate showed that AFP level was also an independent risk factors of RFS (HR 2.688; 95% CI 0.922–7.836; *P* = 0.070). Moreover, pT stage is the other independent risk factor (HR 1.894; 95% CI 1.066–3.366; *P* = 0.029).

Considering the substantial overlap of patients in AFP and CA19–9 analyses, we put the indicators, AFP level and CA19–9 level, together to perform multivariate analyses in order to eliminate the eclipsing influences and ensure the objectivity of the consequence. Considering the pre-operative AFP testing was not universal, we sheltered AFP from multivariate analyses and found that CA19–9 could be a fair substitute to predict the OS (HR 7.816; 95% CI 2.084–29.308; *P* = 0.002) as well as RFS (HR 4.386; 95% CI 1.824–10.547; *P* = 0.001) when AFP data was unavailable. The univariates and multivariate analyses outcomes were showed on Tables 3 and 4.

**Table 3:**
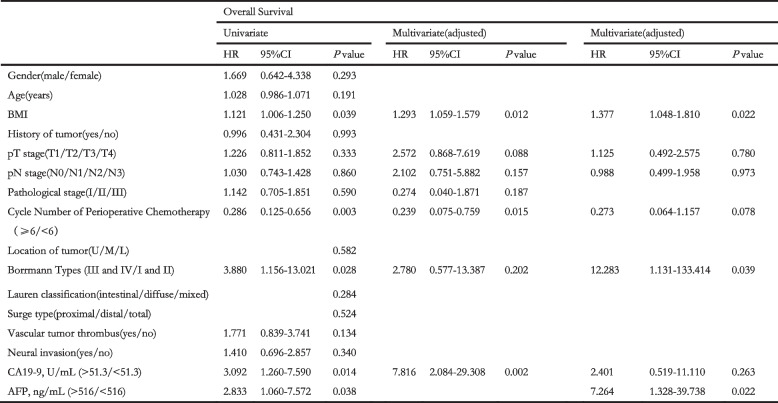
Univariate and multivariate analyses to identify the risks of overall survival

**Table 4:**
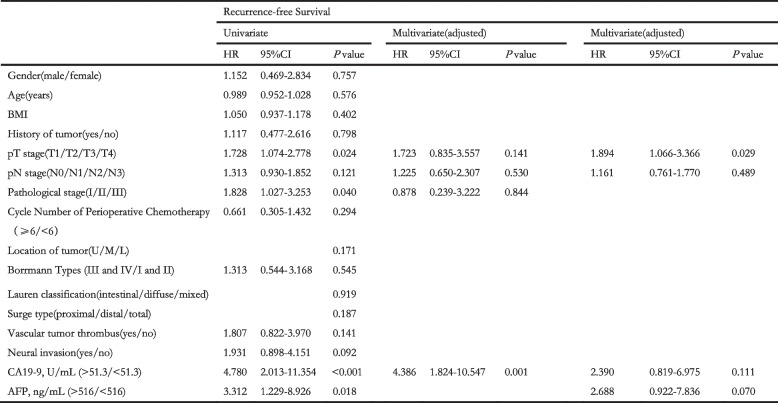
Univariate and multivariate analyses to identify the risks of recurrence-free survival

## Discussion

This is a single-center retrospective study exploring the relationship between preoperative serum tumor markers and HAS patients’ prognosis. As a rare type of gastric cancer, the diagnosis criteria of HAS was based on a small scale of case reports [12]. In other words, the evidence-based clinical guidelines are still lacking so far. In this study, we have adopted the primitive diagnosis criteria—the area of hepatocellular differentiation was found in the tumor tissue, no matter the level of AFP value—as the selection standard [13]. We acknowledge that the elevated serum AFP is an important trait of HAS, but high serum AFP should not to be the diagnosis criteria based on current evidence. Actually, Qu et al. found that 84.30% HAS patients presented with high AFP values (> 40 ng/mL) [16]. In our study, however, the high AF*P* values (> 40 ng/mL, as above mentioned) was account for 34.9% (37/106) which was obviously lower than previous studies. This result may attribute to the relative early stage of tumor in our study, whereas most of patients with HAS in the real world were advanced stage and lost chance for radical surgery. However, the pathological stage, in our study, didn’t show a significant difference between the low AFP group and the high AFP group (P value = 0.640). The underlying reason maybe that we didn’t included the advanced stage HAS patients and the data of high AFP group was not sufficient.

We have demonstrated that high AFP in preoperative serum was an independent risk factor for OS and RFS of HAS patients. Though the serum AFP elevating mechanism of HAS was still ambiguous and the opinions varied [3, 17], we speculate that serum AFP level reflected the HAS progression and may be involved with tumorigenesis. In fact, Zhu et al. has found that serum AFP play a critical role in the initiation of liver cancer stem cell after analyzing PI3K/AKT signal pathway [18]. The essential molecular mechanism underling the high serum AFP and poor survival of HAS required further research.

We also detected that preoperative CA19–9 level was associated with the HAS patients’ prognosis in multivariate analyses. However, no significant difference was observed for OS or RFS in the multivariate co-analyses with AFP. Considering the difficulty of biopsy diagnosis for HAS and the serum AFP test was not universal for gastric cancer in most medical center, CA19–9 could function as a valuable prognostic index for HAS patients. In other words, CA19–9 could perform as a fair substitute to predict the HAS patients’ prognosis. By the way, we found that CA19–9 level was strongly affected by Borrmann type. In our study, all 11 high-level CA19–9 cases were Borrmann III or IV types. Similar results were not observed in AFP cases. Further study should be implemented to identify this characteristic. Other common clinical tumor makers like CEA, CA24–2 and CA72–4 failed to demonstrate any statistical significance in the study.

Interestingly, pathological stage had not shown prognostic significance for OS (adjusted HR 0.274; 95% CI 0.040–1.871; *P* = 0.187) or RFS (adjusted HR 0.878; 95% CI 0.239–3.222; *P* = 0.844) in the relative multivariate analyses and the HR was less than 1 which went against common sense. We thought the pathological stage data functioned as statistic noise in multivariate analyses. The reason might be the innate selection bias or the limitation of sample size. Therefore, we eliminated the “pathological stage” from the later multivariate analyses.

The results also indicated the significance of perioperative therapy. Compared to received less than 6 cycles perioperative chemotherapy, the HAS patients who received more than 6 cycles of perioperative chemotherapy trended to achieve better survival (OS: adjusted HR 0.212; 95% CI 0.044–1.027; *P* = 0.054), which were similar with the gastric adenocarcinoma [11]. Moreover, we found that BMI was closely related with patients’ overall survival. There were two main speculations. Firstly, our OS statistic index reflected all-cause mortality and lower BMI population were inclined to have healthier lifestyle.

In the previous work, HAS patients assumed worse prognosis than gastric adenocarcinoma regardless of serum AFP level [3]. Liu et al. studied 45 patients diagnosed as HAS and found the 5-years survival rates of HAS were 9% compared non-HAS 44% [19] . The survival outcomes of HAS patients in our research were much better than the previous studies. We deduced that patients in our study all underwent radical surgery resulting in the superior prognosis. Actually, Sun et al. analyzed 26 HAS patients and suggested that an early stage and radical surgery might lead to a better prognosis [20].

The employment of X-tile tool which used the minimum *P* value method from the log-rank χ 2 statistics in our study enhanced the scientific soundness to a large extent [15]. The upper-limit of serum AFP values 7 ng/mL was determined by the clinically normal population and this value, obviously, was not qualified to be the cut-off point. In the past studies, researchers usually identified the cut-off value arbitrarily. Wang et al. dichotomized 24 HAS patients by the cut-off value of 500 ng/mL in 2019 [5]. Lin et al. divided the patients by 20 ng/mL and 300 ng/mL in 2014 [21]. The limitations of sample size make it difficult for the previous researchers to split the patients using a methodologic approach. In the study, the AFP and CA19–9 cut-off value calculated by X-tile were equal by accident. The reason might be the limitation of the sample size. Another explanation was that the survival of HAS patients went worse prominently from a tiny range.

Undoubtedly, this study also has some limitations. Firstly, this is a retrospective study of a single center and the possibility of selection bias is inevitable. Further prospective research studies are required to certify our results. Secondly, the post-operative therapeutic methods of HAS varied and the influence couldn’t be assessed for the treatment details unavailable. This might impair the credibility of the results. Thirdly, we didn’t include HAS patients at advanced stage while the HAS patients in the real world usually diagnosed with metastases. Although the study is just the tip of the HAS iceberg, the superior survival has pointed the brilliant way for the future treatment that diagnosed at an early stage, receiving radical surgery and undergoing systematic peri-operative therapy will breeds favorable survival outcomes.

## Conclusion

Preoperative serum AFP level is qualified to predict the HAS patients’ prognosis and preoperative serum high AFP is an independent risk factor for OS and RFS of HAS patients. Preoperative serum CA19–9 could be an alternative choice when AFP is unavailable.

## Data Availability

The datasets used and analyzed during the current study are available from the corresponding author on reasonable request.

## Abbreviations

HAS: hepatoid adenocarcinoma of stomach
AFP: alpha-fetoprotein
OS: overall survival
RFS: recurrence-free survival
CA: Carbohydrate antigen
HCC: hepatocellular carcinoma
CEA: Carcinoembryonic antigen
CT: Computed tomography
NCCN: The National Comprehensive Cancer Network
SD: standard deviation
IQR: interquartile range.
